# Bioinspired material architectures from bighorn sheep horncore velar bone for impact loading applications

**DOI:** 10.1038/s41598-020-76021-5

**Published:** 2020-11-03

**Authors:** Trevor G. Aguirre, Luca Fuller, Aniket Ingrole, Tim W. Seek, Benjamin B. Wheatley, Brett D. Steineman, Tammy L. Haut Donahue, Seth W. Donahue

**Affiliations:** 1 Department of Mechanical Engineering, Colorado State University, 400 Isotope Drive, Fort Collins, CO 80521 USA; 2 Department of Biomedical Engineering, University of Massachusetts, Amherst, MA 01003 USA; 3 Mechanical Engineering Department, Bucknell University, Lewisburg, PA 17837 USA; 4 Department of Biomechanics, Hospital for Special Surgery, New York, NY 10021 USA

**Keywords:** Mechanical engineering, Bioinspired materials, Mechanical properties

## Abstract

Rocky Mountain bighorn sheep rams (*Ovis canadensis canadensis*) routinely conduct intraspecific combat where high energy cranial impacts are experienced. Previous studies have estimated cranial impact forces to be up to 3400 N during ramming, and prior finite element modeling studies showed the bony horncore stores 3 × more strain energy than the horn during impact. In the current study, the architecture of the porous bone within the horncore was quantified, mimicked, analyzed by finite element modeling, fabricated via additive manufacturing, and mechanically tested to determine the suitability of the novel bioinspired material architecture for use in running shoe midsoles. The iterative biomimicking design approach was able to tailor the mechanical behavior of the porous bone mimics. The approach produced 3D printed mimics that performed similarly to ethylene–vinyl acetate shoe materials in quasi-static loading. Furthermore, a quadratic relationship was discovered between impact force and stiffness in the porous bone mimics, which indicates a range of stiffness values that prevents impact force from becoming excessively high. These findings have implications for the design of novel bioinspired material architectures for minimizing impact force.

## Introduction

High-energy impact causes substantial damage to structures (e.g., vehicles) and humans (head and joints). Rocky Mountain bighorn sheep (*Ovis canadensis canadensis*) routinely experience repetitive high energy cranial impacts for up to several hours per day during mating season, which lasts several weeks^1^. During impact, the horn experiences forces of up to 3400 N^2^, and bending stresses ranging from 1 to 6 MPa in tension and 1 to 7 MPa in compression^3^. After impact, the ram may seem momentarily stunned but otherwise show no long term ill effects from ramming^1^. The keratinous horn material has been reported to have high work of fracture to prevent catastrophic failure during loading^4^. To supplement the outer keratin layer, the bony horncore has been shown to play a large role in energy absorption during simulated quasi-static^5^ and dynamic^6^ loading conditions and reduce brain cavity accelerations during impact^6^. The unique architecture of the horncore is made up of a foam-like bone structure composed of sail-like features (i.e. velar bone), which differs from the more rod-like structure of trabecular bone^7^. Trabecular architecture is typically characterized by trabecular thickness, spacing, and number, connectivity density, and bone volume fraction. Analogously, velar bone can be characterized by velar thickness, spacing, and number, connectivity density, and bone volume fraction. Interestingly, velar bone has a volume fraction comparable to typical trabecular bone (approximately 20%), but individual velae have a thickness of 2.87 ± 0.78 mm, which is approximately 26 times higher than typical trabecular bone struts^7^. There are also about 20 times fewer velae per unit length compared to trabeculae, and the separation between velae is about 20 times greater than the separation between trabeculae. Due to the extreme impact forces generated during ramming, these differences suggest that velar bone architecture may be evolutionarily adapted to store energy during dynamic loading to prevent brain damage. Conversely, human head impacts often result in traumatic brain injury (concussions)^8^ and chronic traumatic encephalopathy^9,10^. To help design head trauma prevention materials and mechanisms, researchers have begun to study bighorn sheep keratin horn and bony horncore to better understand the energy absorption and storage capabilities of these materials. The impact properties of horn keratin^11^ and other horn-like structures^12^ have been studied, but these results have yet to be applied to developing a bio-mimicked material or structure. Bio-mimics for armor and structural applications have been successfully developed for natural impact resistant and energy-storing materials such as nacre^12–18^, mantis shrimp dactyl club^19,20^, woodpecker skull^21^, conch shell^22^, and beetle shell^23^, but not bighorn sheep horns. Thus, mimicking the velar bone architecture may lead to novel structures optimized for weight-efficient energy storage for impact applications.

Athletic footwear (e.g., running/tennis/basketball shoes and hiking/climbing/military boots) have a variety of purposes and mechanical needs for effective and optimal performance, but all have impact in common. Running generates vertical ground reaction forces of 2.5–3 × bodyweight^24,25^, and joint reaction forces of 3.6–4.2 × bodyweight in the knee^26,27^, and as high as 10 × bodyweight in the hip^28^. The ground reaction force may be up to 4.6 × bodyweight for moderate impact jumping^29^ and up to 11.6 × bodyweight during higher impact jumping^30^. These impact forces are exacerbated in military personnel whose effective body mass is higher because of additional gear (~ 22 kg)^31^. These high forces from physical activity have been associated with tibial stress fractures^32^, damage to soft tissues^33^, and running-related injuries costing between $28.8 and $37.2 billion annually to individuals and insurance companies in the United States^34^. It has been hypothesized that running-related injuries can be reduced if shoes are better designed to (1) limit excessive forces, (2) support the foot during standing, and (3) guide the foot to the ground^35^.

Running shoe midsoles have traditionally been made from ethylene–vinyl acetate (EVA) because of its durability and low density^36^ and resistance to degradation^37^. More recently, polyurethane foam has been used in running shoe midsoles because of its long term mechanical properties (low creep)^38,39^. EVA foams are typically made through traditional foaming techniques where a physical or chemical blowing agent creates gas pockets that produce a random closed-cell architecture^40^. Typical cell sizes in these stochastic foams are on the order of 7–11 μm^41^. It has been shown that the average cell size and uniformity of the cells (distribution and size) are two important parameters to control for mechanical property enhancement^41^. For impact applications, the primary mechanical properties of interest are the maximum impact force and the energy storage. During typical impact tests performed per ASTM F1976^42^ on EVA foams, the maximum impact force is 985–992 N and the energy stored is 2–7 J^43^. That study used whole shoes (size 8.5 US) but only the heel was subject to impact. Measured under a variety of testing methods and shoe types, midsole stiffness was found to be between 30–439 N/mm^38,43–45^. These studies suggest that lower stiffness midsoles provide better cushioning (i.e. more energy stored) but experience high impact forces because the foot is not slowed down fast enough. These findings imply that there is a balance between midsole compliance and impact force minimization.

We hypothesized that bighorn sheep velar bone architecture could be mimicked to improve the impact performance of athletic footwear midsole structures by increasing energy storage and reducing impact force, and thus, reduce the risk of injury. These mimicked structures were analyzed using finite element models, fabricated via additive manufacturing, and mechanically tested using quasi-static compression and dynamic impact tests, then compared to commercially available running shoe midsole materials.

## Materials and methods

### Material samples

**Figure 1:**
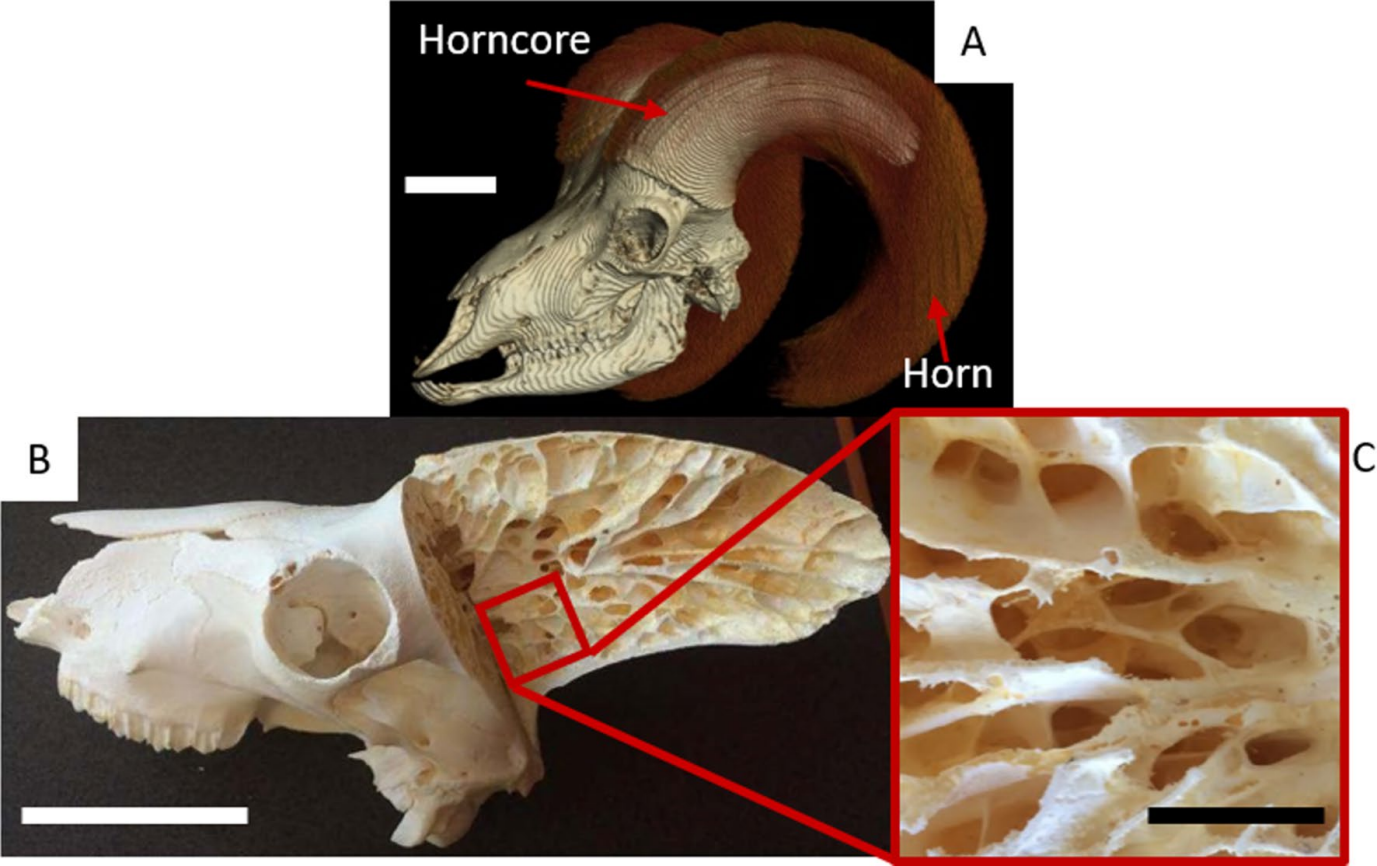
(**A**) Horn and horn core spatial arrangement, (**B**) horn core longitudinal-section showing the velar bone inside the thin cortical shell, (**C**) velar structure in the compressive region of the horncore. The scale bar in images (**A**) and (**B**) are 10 cm and the scale bar in image (**C**) is 2.5 cm.

Velar architectures were obtained from five male bighorn sheep skulls, which were provided for research purposes by the state of Colorado Department of Natural Resources under Colorado Parks and Wildlife scientific collection license number 14SALV2052A2. The skulls were obtained from sheep that were killed by motor vehicle accidents and frozen shortly after death. Thus, Colorado State University’s Research Integrity and Compliance Review Office determined the research was exempt from Institutional Animal Care and Use Committee oversight. The age of the bighorn sheep was unknown at the time of collection but was estimated by measuring the length of the horn^46^. Sheep horn curl lengths measured between 70 to 95 cm, which gives an age range between five to nine years, which is old enough to be considered mature^47^. The skulls were scanned using a Gemini Time-of-Flight Big Bore PET/16 slice CT scanner (Philips Healthcare, Andover, MA, USA). Scan voltage was 140 kV, current was 321 mA, and time was exposure 350 mAs, which produced cubic voxels with edge length 0.73 mm/pixel. Since the velar architecture is much larger than trabecular architecture it is worth noting that CT scan resolution used in our study exceeds the suggested minimum resolution for accurate imaging^48^. The architectures for the velar bone mimics (VBMs) were created from the left horn from five different sheep. Sections of the velar architecture were cropped from the regions of high compressive stress in the horncore^6^. These regions were chosen for the mimics because running shoes experience compressive loading during standing and gait. The region of interest (ROI) for each horncore was a 45 mm cube, which maximized the amount of velar bone that could consistently be utilized from the compressive region of the horncore from each sheep. Bighorn sheep velar architecture and the velar bone ROI are shown in Fig. 1.

### Velar bone architecture

**Figure 2:**
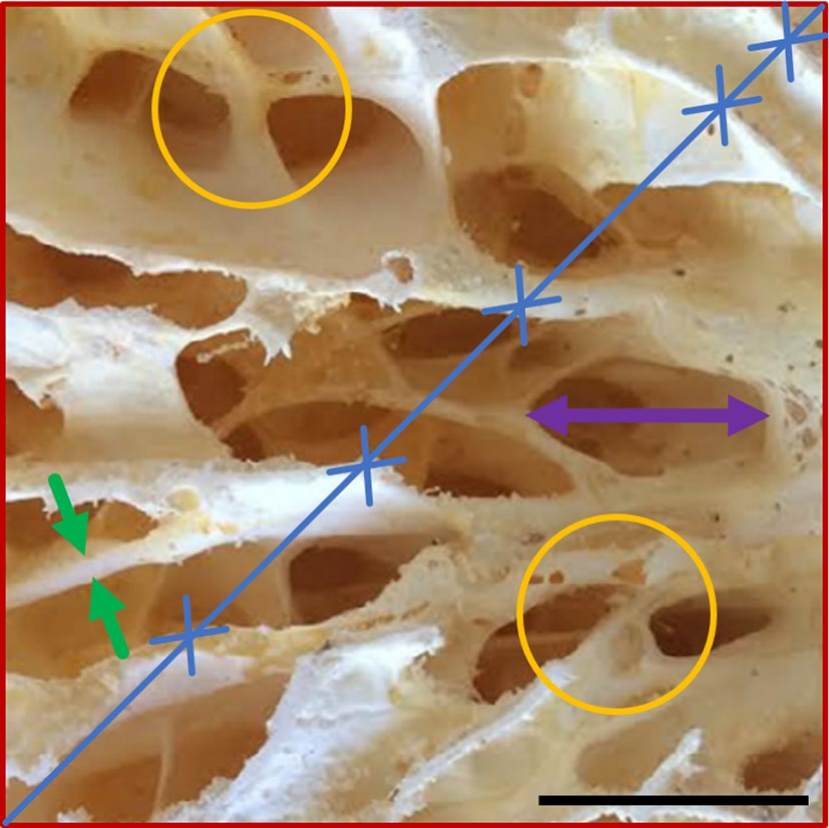
Velar bone architectural indices: velar thickness, V.Th (green arrows), velar separation, V.Sp (purple arrow), velar number, V.N (blue lines and crosses), TV (red square), and Conn.D (orange circles). The scale bar is 2.5 cm.

Velar bone architectural index measurements are depicted in Fig. 2 and were measured using BoneJ^49^. The velar bone volume fraction (BV/TV) is the volume of bone (BV) normalized by the total volume (TV) of the velar bone ROI. The velar thickness (V.Th) is the average thickness of all velae within the ROI. The velar separation (V.Sp) is the average linear distance between two velae. The velar number (V.N) is the number of velae per unit line length. The connectivity density is the total number of connections between two or more velae normalized by the volume of the ROI.

### Bighorn sheep velar bone mimic generation

**Figure 3:**
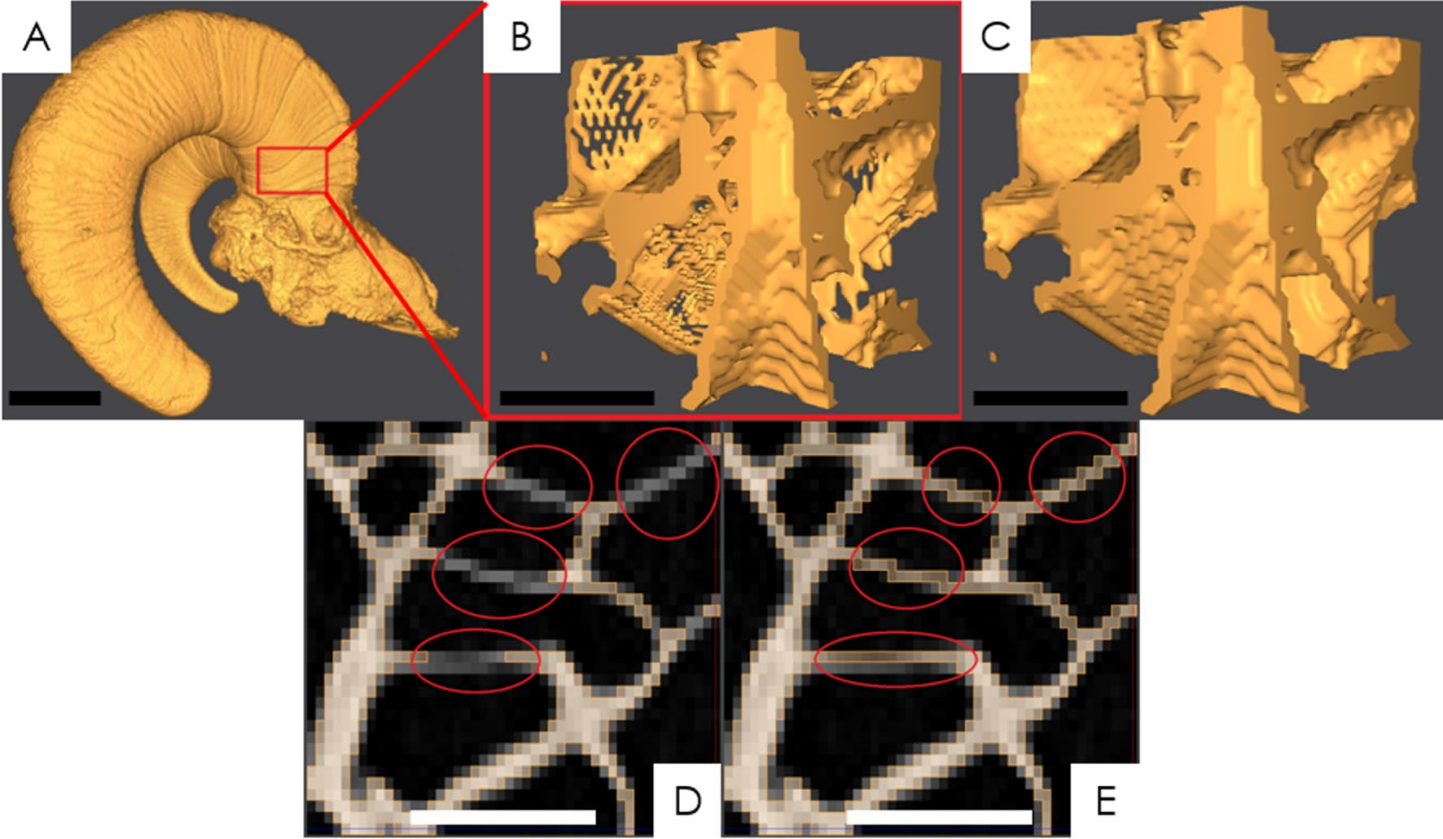
(**A**) Binarized ram skull, (**B**) velar cube cropped from the compressive region of horncore indicated in (**A**), (**C**) repaired velar cube, (**D**) velae perforations in threshold mask layer, (**E**) repaired perforations in the threshold mask layer. For image (**A**) the scale bar is 50 mm and for images (**B**)–(**E**) the scale bar is 45 mm.

After velar bone architecture was quantified, each ROI was cropped out of the CT scans to generate 3D models of the velar bone mimics (VBMs). First, Seg3D (version 2.2.1, University of Utah, Salt Lake City, UT, USA) was used to separate the bony horncore and horn keratin using manual binary thresholding operations (Fig. 3A). Flawless global segmentation of the horn and horncore was difficult due to contrast differences in the images that compose the DICOM files. As a result, small perforations in the velar structure were inevitable and needed minor repair (Fig. 3B). These perforations were repaired by manually adding pixel values to the threshold mask layer (Fig. 3D,E). CT images of each ROI were only repaired in CT scan regions where it was apparent that bony material was displayed within the CT images yet there were no pixel values in the threshold mask layer (Fig. 3D). Finally, the repaired velar structure (Fig. 3C) was saved in the ASCII STL file format for further mimic preparation.

**Figure 4:**
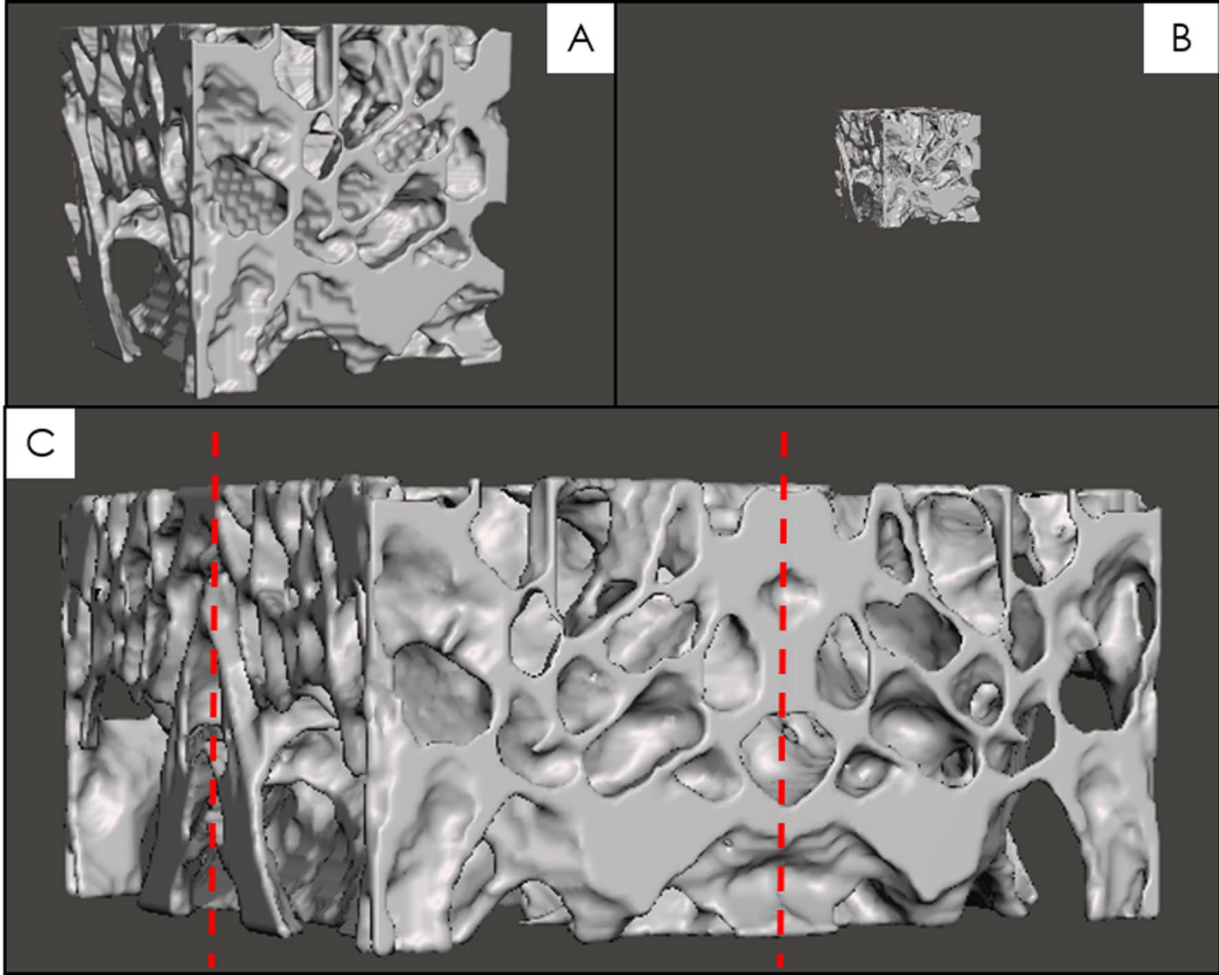
(**A**) 45 × 45 × 45 mm unit cell cube, (**B**) 20 × 20 × 20 mm scaled cube, (**C**) 40 × 40 × 20 mm velar bone mimic structure. The dashed red lines in (**C**) indicate lines of symmetry.

MeshMixer (version 3, San Rafael, CA, USA) was used to isometrically scale the 45 × 45 × 45 mm cube (Fig. 4A) to produce 20 × 20 × 20 mm unit cell (Fig. 4B), which were then mirrored across two mirror two planes to produce a 40 × 40 × 20 mm geometry (Fig. 4C). Scaling and mirroring provided mimic structures that preserved the natural velar bone architecture and were approximately the same thickness as a running shoe midsole. Additionally, this process allowed us to achieve continuum dimensions using only velar bone from the compressive region of the horncore. For trabecular bone, continuum dimensions have been estimated to be at least five trabecular spacings^50^, therefore we assumed five velar spacings were adequate for the velar bone mimics. These mimicked geometries were exported in the ASCII STL file format for further processing.

**Figure 5:**
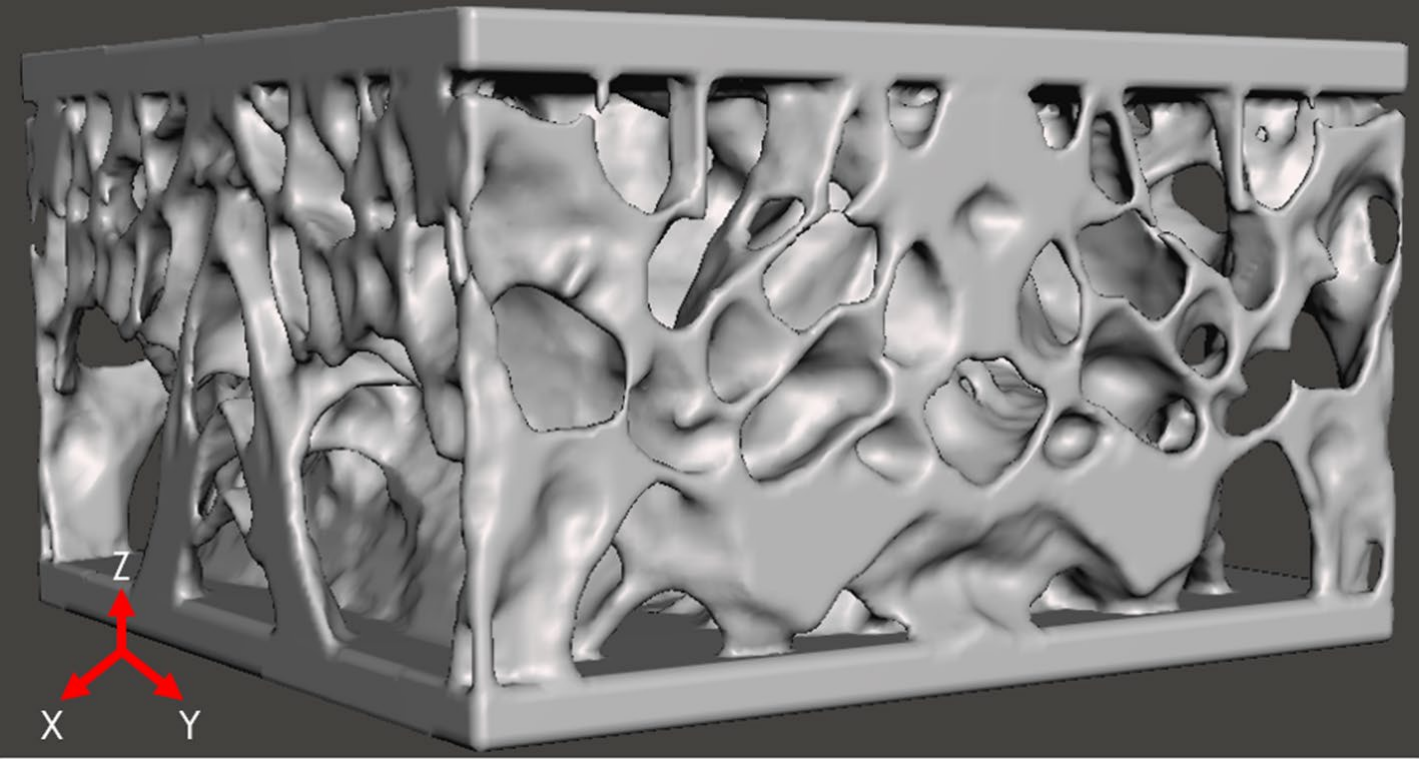
Velar bone mimic sandwich structure after STL file repair.

NetFabb (Autodesk, San Rafael, CA, USA) was used to further repair the mimic STL files using automated operations to fix errors during the surface triangulation process (i.e. remove duplicate and penetrating faces). In this step, a 2 mm thick plate was added to the top and bottom to create a sandwich structure (Fig. 5). The plate was added so there would not be free-floating struts to better approximate the boundary conditions that the velar architecture would experience in-vivo during loading and how the bioinspired mimics would be incorporated into shoe soles. After repair, the final files were exported in the ASCII STL file format to be used in mesh generation for the FEA study and additive manufacturing.

### Manufacture and mechanical testing

The novel velar bone mimics developed in section “Bighorn sheep velar bone mimic generation” were 3D printed on a Carbon Speedcell™ using Elastomer Polyurethane (EPU) #40. Mimics were manufactured by Ramaco Carbon (Sheridan, WY, USA) and printed with 75-μm resolution in the x–y plane and 100-μm layer thickness where the build direction was coincident with anatomical loading during impact (z-direction Fig. 5). The EPU #40 had an elastic modulus of 6.81 MPa, Poisson’s ratio 0.48, and material density 1.025 g/cc. For comparison to the velar architecture, three running shoe EVA foams were tested. EVA had an elastic modulus 25 MPa, Poisson’s ratio 0.48, and material density 0.965 g/cc^51^. Running shoe midsole high-density (HD), medium-density (MD), and low-density (LD) EVA foam samples were mechanically tested to compare to the EPU VBMs. Seven coupons of each EVA foam and each VBM were used for both static and dynamic tests. All test coupons were 40 × 40 × 20 mm. Quasi-static compression was used to assess the mechanical behavior of the EVA foams and EPU VBMs under compression, which would be experienced by a shoe sole during standing. Quasi-static compression tests were performed using a hydraulic load-frame (Instron model 8501, Norwood, MA, USA) in displacement control per ASTM F1621^52^. Crosshead displacement rate was 5 mm per second, samples were compressed to 25% strain (5 mm), and then released at 5 mm per second. This displacement was chosen as the maximum allowable displacement to ensure runner comfort^53^. Applied load and crosshead displacement were measured and used to compute energy storage, specific energy storage, and stiffness. For the compression tests data were collected at 100 Hz. Dynamic impact tests were performed to assess the mechanical behavior of the EVA foams and the EPU VBMs during impact, which would be experienced by a sole during running. Dynamic impact tests were performed on custom drop tester inspired by the design presented in ASTM F1976^42^. The mass of the missile was 8.5 kg and was dropped from a height of 60 mm to provide an energy of 5 J at impact. Missile position was measured using a linear displacement transducer (176-0521-L3N, Firstmark Controls, Creedmoor, NC) and impact force was measured using an impact force transducer (200B05, PCB, Depew, NY). Impact force and displacement were used to compute the maximum impact force, energy storage, and specific energy storage. For the impact tests data were collected at 5000 Hz.

Energy storage ($E_{S}$), during quasi-static compression and impact, was computed by numerical integration of the force–displacement loading curve using Eq. (1).

1 $$E_{S} = \int_{0}^{{\delta_{\max } }} {F\;d\delta }$$

where *F* is the applied force and $\delta$ is displacement.

Due to differences in the volume of material present in each velar bone mimic and EVA foam, the specific energy storage was computed using Eq. (2)^54^.

2 $$W_{s} = \frac{{\mathop \smallint \nolimits_{0}^{{\epsilon_{max } }} \sigma (\varepsilon )\;d\varepsilon }}{{\Delta \rho \rho_{s} }}$$

where $W_{s}$ is the specific energy storage, is the max strain $\epsilon_{max}$, $\sigma (\varepsilon )$ is the stress at each value of the strain, $\Delta \rho$ is the relative density of the foam (BV/TV is equivalent), and $\rho_{s}$ is the density of the solid material that the foam is comprised of.

### Finite element model generation

Velar bone mimics were meshed in ICEM CFD (version 18.1, ANSYS, Canonsburg, PA, USA) to generate a linear triangular shell (S3) mesh and analyzed using Abaqus FEA (Dassualt Systems, Vélizy-Villacoublay, France, EU). Shell elements were chosen because these elements can accurately model the behavior of cellular solids in finite element models^54^. Shell element thickness varied between each velar bone mimic due to intrinsic differences in velar thickness between animals but did not vary within an individual finite element model. Quasi-static compression was simulated for each structure by placing the velar bone mimics between two rigid plates, applying an encastre boundary condition to the bottom plate, and allowing the top plate to translate in the z-direction only (Fig. 5). From the starting configuration, the top plate was displaced at 5 mm per second for a total displacement of 5 mm to simulate the mechanical testing procedure. All finite element models used linear elastic material properties. For each velar bone mimic, the shell thickness was iterated until the simulated stiffness closely matched the experimentally measured stiffness^55^. Self-contact was used to capture the behavior of contact between locally buckled velae. In Abaqus, the interaction property was set to “ALL WITH SELF” using the general contact option. The tangential behavior was set with a friction penalty of 0.2 and normal behavior was set to hard contact^54^. To reduce computation time mass scaling was utilized; thus, the Dynamic/Explicit solver was used. To avoid small vibrations (oscillatory behavior) in the force–displacement curves caused by mass scaling, minimal damping was used (α = 1 × 10^–5^)^56^. Models were given the experimentally determined properties of EPU 40 (E = 6.81 MPa and ν = 0.48). Optimal mesh density was determined via a numerical convergence study^57,58^. Five unique mesh densities ranging from 13 to 222 elements per cubic millimeter were created for VBM3, which had the smallest average velar thickness. To determine whether the mesh had converged, the change in strain energy between each mesh was analyzed and compared to the finest mesh as a percent difference using Eq. (3).

3 $$\Delta = \frac{{X_{N} - X_{i} }}{{X_{N} }}100\%$$

where Δ is the percent difference and X is strain energy. X_N_ is strain energy for the finest mesh in the mesh convergence study and X_i_ is the strain energy for the other meshes used in the study. Convergence was achieved at a mesh density of 188 elements per cubic millimeter, which had a 2.16% difference from the finest mesh density of 222 elements per cubic millimeter.

### Velar bone mimic iterative design process

**Figure 6:**
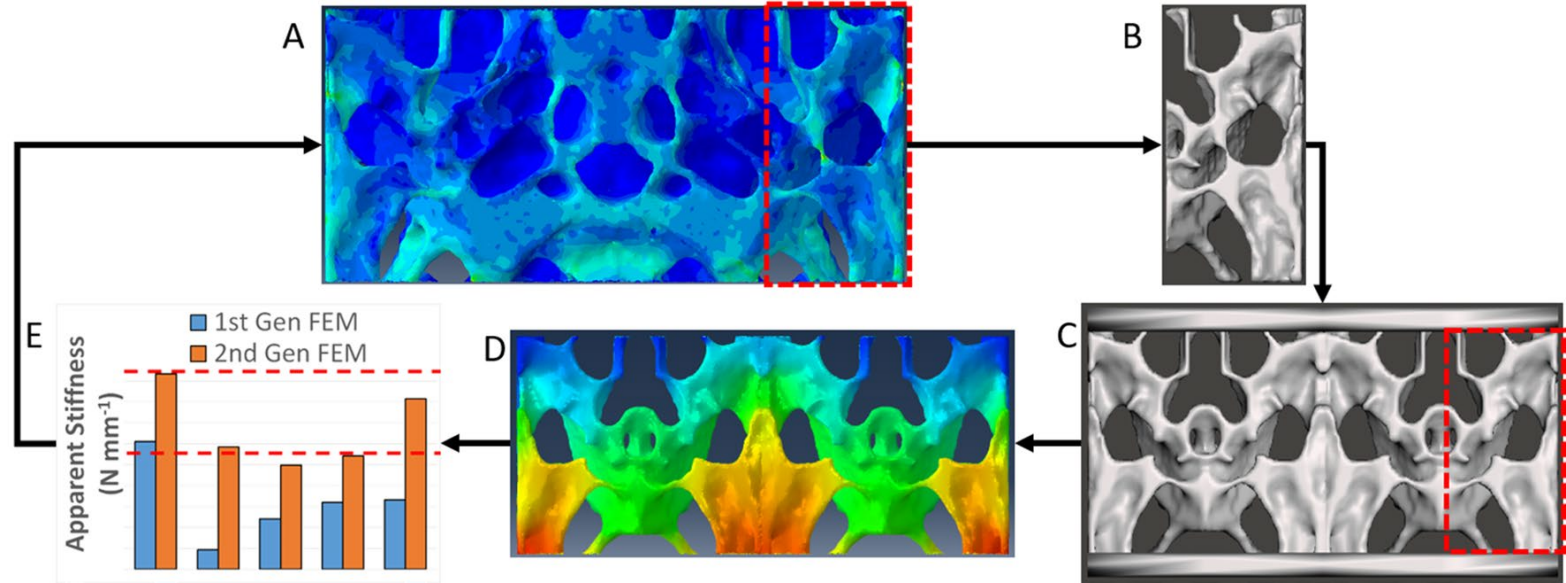
(**A**) Unit cell encompassing regions with highest energy storage, (**B**) 2nd generation unit cell, (**C**) 2nd gen VBM coupon, (**D**) finite element model, (**E**) experimental quasi-static compression results; dashed red lines indicate upper and lower EVA stiffness constraints.

After validating the finite element models with data from the quasi-static compression tests of the first-generation velar bone mimics, an iterative design process was used to improve the mechanical performance of the VBMs. The goal was to increase energy storage, reduce impact force, and satisfy the self-imposed stiffness requirements. We chose the average quasi-static stiffness values, from the mechanical tests, of the HD (47.48 N/mm) and LD (23.91 N/mm) EVA foams as the upper and lower stiffness limits for the second generation VBMs. The iterative design process is depicted in Fig. 6. The procedure is explicitly described in the following five steps. (1) First-generation VBM finite element models were visually interrogated, in Abaqus, to identify regions with the highest strain energy storage, as reported by the Abaqus strain energy color maps (Fig. 6A). (2) These regions with the highest energy storage were visually correlated back to the original unit cell STL file and then isolated from within the original unit cell (Fig. 6B). (3) The new unit cell was then used to construct the second generation of velar bone mimics (Fig. 6C). (4) Second-generation VBMs were then subjected to the same finite element modeling procedures as the first-generation VBMs (Fig. 6D). (5) Stiffness of the second-generation VBM finite element models were measured and compared to the EVA foam stiffness constraints (Fig. 6E). This process was iterated until the second-generation VBMs stiffness were within the range of the EVA foam stiffness constraints, at which time the process was terminated. The number of iterations to achieve VBM-2G specimen that were within these stiffness constraints was between one and three iterations. These second-generation mimics were named as VBM-2G and unit cell size ranged from 32–256 unit cells per mimic.

### Statistical analyses

The velar bone architectural indices (BV/TV, V.Th, V.Sp, V.N, and Conn.D) were compared to trabecular bone architectural indices from the distal femur and proximal tibia of human^59^ and sheep^59–63^. These anatomical locations were chosen because they experience impact loading during physical activities such as running and jumping. Analysis of variance (ANOVA, α = 0.05) was used to compare the porous bone architectures of each species. Stepwise regressions (α = 0.05) were used to determine the influence of velar architecture on mechanical performance of the VBMs during the compression and impact tests. For the stepwise regressions, the candidate independent variables were V.Th, V.Sp, and Conn.D measured from the velar bone mimics. V.N and BV/TV were excluded from the regression models to avoid collinearity since they are both correlated with V.Th, V.Sp and Conn.D. For the quasi-static compression tests, the dependent variables were stiffness, energy stored, and specific energy stored. For the impact tests, the dependent variables were the maximum impact force, energy stored, and specific energy stored. ANOVAs (α = 0.05) were used to compare the VBMs and EVA data from the impact and compression tests. Since our goal was to make VBMs comparable to commercially available running shoe midsole material (EVA), we followed up ANOVAs with Bonferroni’s test to compare each EVA group (LD, MD, and HD) with each first and second generation VBM. The stepwise regressions and ANOVA were performed in Minitab (version 18, State College, PA, USA). Linear regressions were performed on the impact force and stiffness for EVA foams, the first-, and second-generation VBMs. For the second-generation VBMs two linear regressions were performed for values lower than 60 N/mm or greater than 80 N/mm due to the differences in slopes. Additionally, a quadratic regression was performed between impact force and stiffness for the first- and second-generation VBMs combined.

## Results

### Velar architecture quantification

Velar architectural index measurements from the horncore ROI are presented in Table 1. Velar bone volume fraction showed no difference from human^59^ (27.56 ± 8.9%, p = 0.992) and sheep^59–64^ (30.60 ± 8.04%, p = 0.851) trabecular bone volume fraction. Velar thickness was found to be larger than trabecular thickness in human (0.19 ± 0.05 mm, p < 0.001) and sheep (0.17 ± 0.03 mm, p < 0.001). Similarly, velar separation was larger in than trabecular separation in human (0.57 ± 0.13 mm, p < 0.0001) and sheep (0.47 ± 0.09 mm, p < 0.0001). Velar number was found to be lower than trabecular number in human (1.48 ± 0.33 mm^−1^, p < 0.001) and sheep (1.95 ± 0.39 mm^−1^, p < 0.001). Velar connectivity density was found to be lower than trabecular connectivity density in human (3.56 ± 0.25 mm^−3^, p < 0.001) and sheep (5.76 ± 2.19 mm^−3^, p < 0.001).

**Table 1:** ROI velar architectural index measurements.

Sheep	BV/TV (%)	V.Th (mm)	V.Sp (mm)	V.N (mm^−1^)	Conn.D (mm^−3^)
1	33.14	1.91	5.51	0.19	0.00041
2	21.11	1.40	11.12	0.18	0.00089
3	26.17	1.73	7.05	0.26	0.00068
4	29.72	1.71	6.01	0.29	0.00035
5	30.20	1.67	5.99	0.29	0.00022
Mean ± SD	28.07 ± 4.61	1.68 ± 0.18	7.13 ± 2.3	0.24 ± 0.05	0.00051 ± 0.00027

### Finite element model evaluation

**Figure 7:**
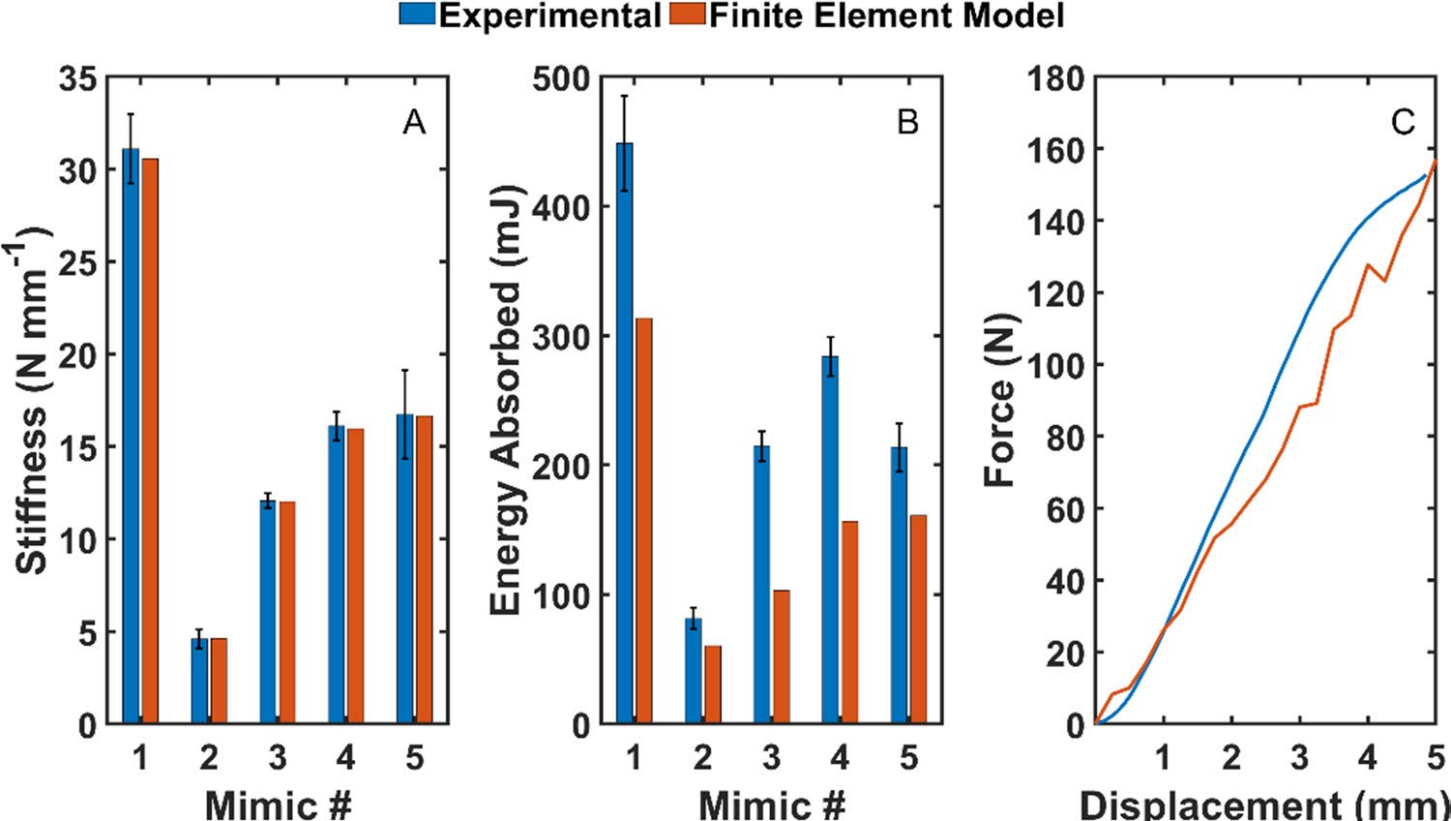
First-generation VBM finite element model evaluation. (**A**) Stiffness, (**B**) energy storage, and (**C**) stress–strain curve comparison between finite element model and mechanical compression tests. The black error bars indicate ± one standard deviation.

Simulated and experimentally measured stiffness showed excellent agreement, where the largest percent error was 0.64% (Fig. 7A). However, simulated energy storage was much lower than the experimentally measured energy storage with differences as large as ~ 60% (Fig. 7B). Energy storage differences can be attributed to differences between the shape of the force–displacement curves (Fig. 7A).

### Finite element modeling of velar bone mimics

**Figure 8:**
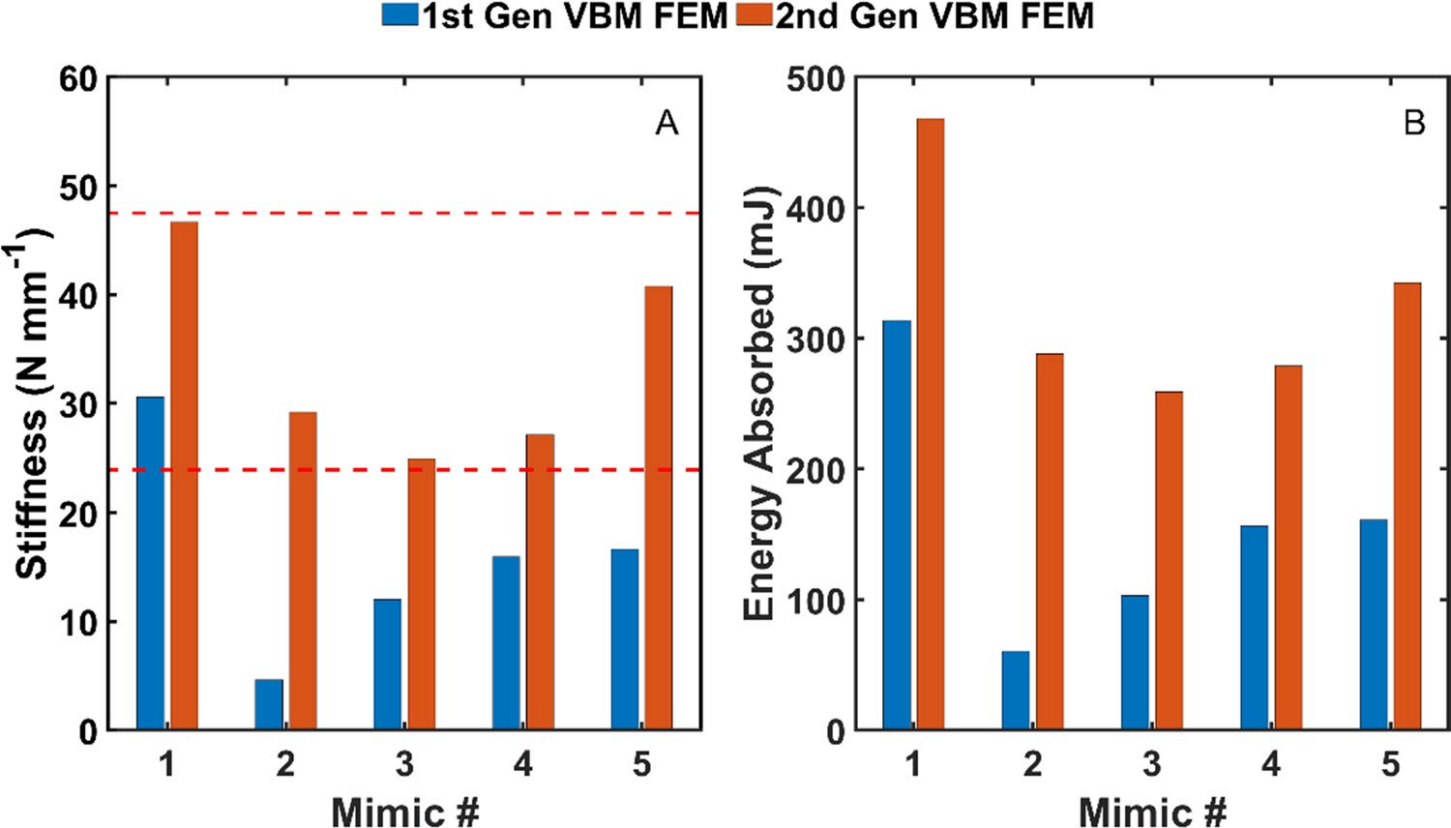
First and second generation VBM finite element model comparison. (**A**) Stiffness and (**B**) energy stored. The dashed lines indicate the EVA foam stiffness constraints.

Shown in Fig. 8 are comparisons of the stiffness and energy storage of the first- and second-generation velar bone mimic finite element models. The results show that after the iterative design process, the second-generation velar bone mimics satisfy the stiffness constraints of the EVA foams and stored more energy than the first-generation velar bone mimics.

### Compression testing

**Figure 9:**
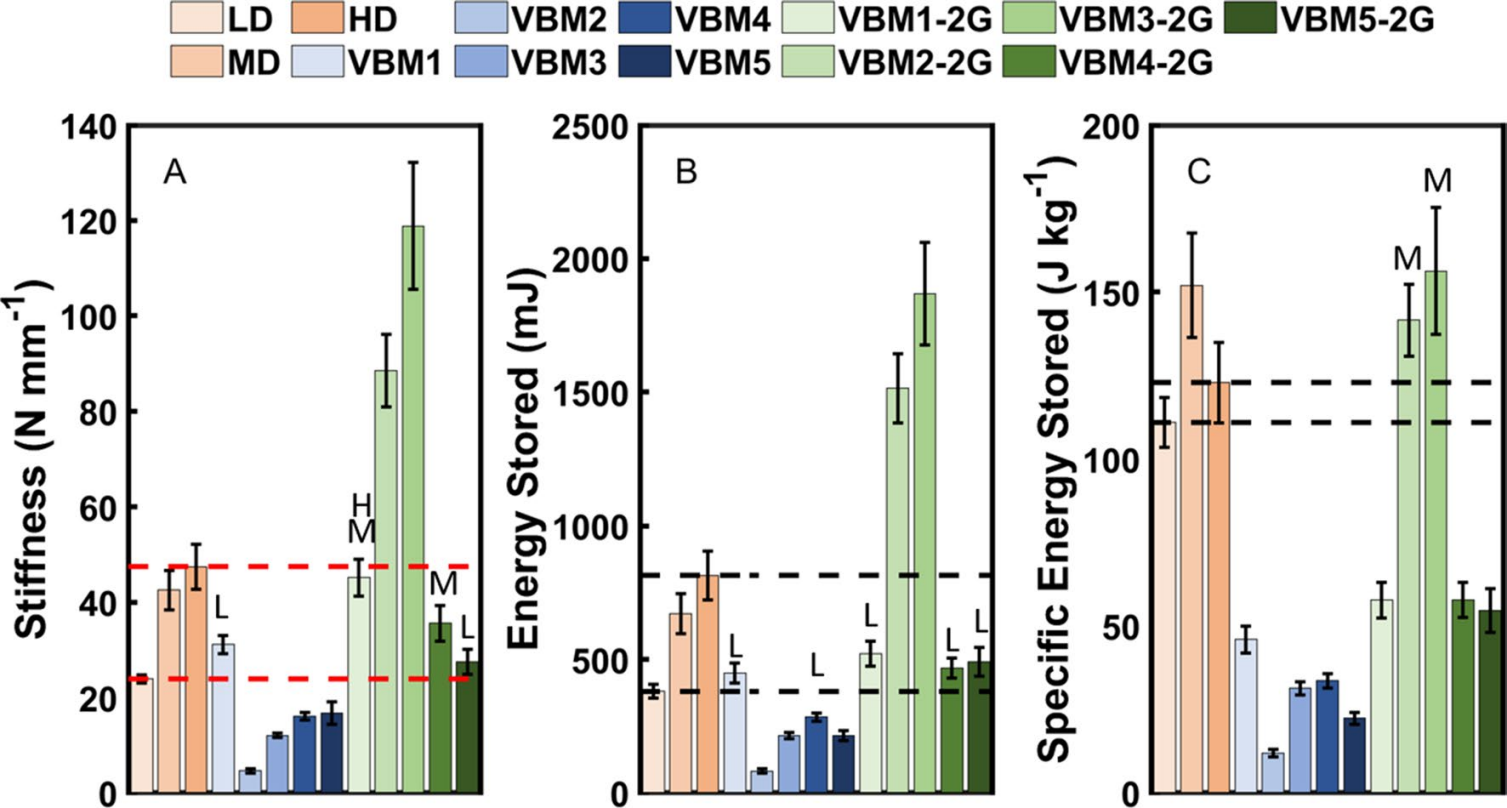
Quasi-static compression test results for the first and second generation velar bone mimics (VBM). (**A**) Stiffness, (**B**) energy stored, and (**C**) specific energy stored. The error bars indicate ± one standard deviation. The dashed lines indicate the ranges for the EVA foams. The red dashed lines in (**A**) indicate the imposed stiffness constraint. L—not significantly different from LD EVA foam, M—not significantly different from MD EVA foam, H—not significantly different from HD EVA foam.

Figure 9 shows stiffness, energy stored, and specific energy stored for EVA foams and velar bone mimics tested in quasi-static compression. Only one of the first generation mimics was within the range of stiffnesses for the EVA foams (Fig. 9A). The second generation mimics met the stiffness constraints in the FEMs; however, the 3D printed coupons were stiffer than the corresponding FEMs. Three of the second generation mimics had stiffnesses that were not different (p > 0.798) from the EVA foams. Several of the first and second generation mimics had energy storage that was not different (p > 0.659) from the low density EVA foam (Fig. 9B). However, only two of the second generation mimics had values of specific energy storage that were not different (p > 0.9) from the medium density EVA foam (Fig. 9C).

### Impact testing

**Figure 10:**
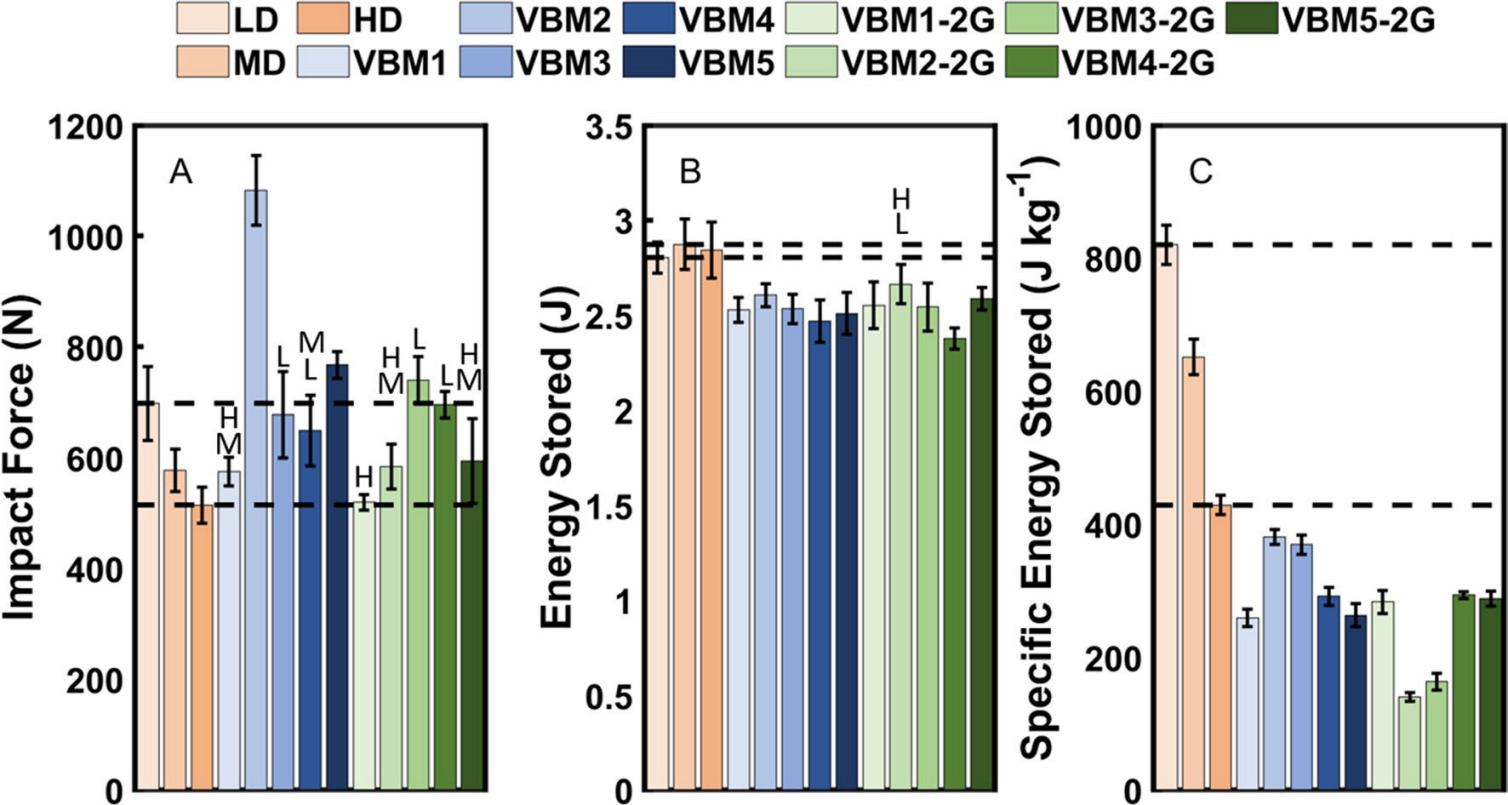
Impact testing results for the first and second generation velar bone mimics (VBM). (**A**) Impact force, (**B**) energy stored, and (**C**) specific energy stored. The error bars indicate ± one standard deviation. The dashed lines indicate the ranges for the EVA foams. L—not significantly different from LD EVA foam, M—not significantly different from MD EVA foam, H—not significantly different from HD EVA foam.

Figure 10 shows impact force, energy stored, and specific energy stored for EVA foams and velar bone mimics tested under dynamic compression. Except one of the first generation mimics, all of the first and second generation mimics had impact forces that were not different (p > 0.702) from the EVA foams (Fig. 10A). However, only one of the second generation mimics had energy storage that was not different (p = 0.110) from the EVA foams (Fig. 10B), and all of the first and second generation mimics had values of specific energy storage that were different (p < 0.001) from the EVA foams (Fig. 10C).

**Figure 11:**
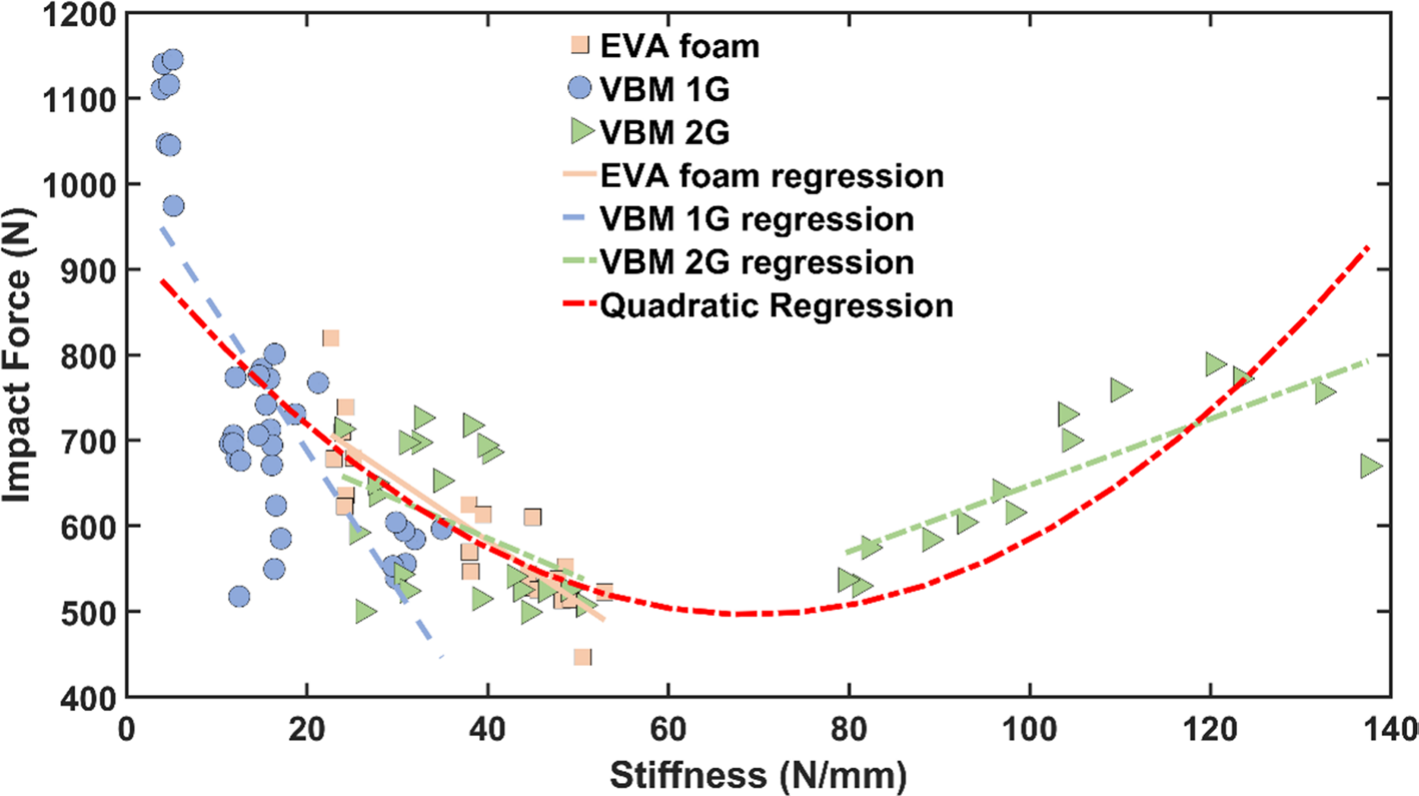
Impact force versus stiffness regressions. First- and second-generation VBM data were grouped together for the quadratic regression.

Figure 11 shows the relationship between impact force and stiffness. Impact force for the EVA foams (R^2^ = 0.743, p < 0.001), first-generation (R^2^ = 0.577, p < 0.001), and the second-generation (R^2^ = 0.178, p = 0.057) VBMs showed negative correlation with stiffness for values of stiffness below 60 N/mm. However, for stiffness above 80 N/mm the second-generation VBMs showed positive correlation with stiffness (R^2^ = 0.670, p < 0.001). Therefore, first- and second-generation VBMs were grouped for a quadratic regression, which showed a significant (p < 0.001, R^2^ = 0.502) quadratic relationship (Fig. 11).

### Stepwise regressions

Strong correlations were found between the velar architectural indices of the VBMs and mechanical testing parameters (Table 2). Mechanical performance was positively correlated with velar thickness for both quasi-static compression and impact. Mechanical performance showed negative correlation during quasi-static compression and positive correlation during impact with both velar spacing and connectivity density.

**Table 2:** Stepwise regression results for compression and impact testing.

Test	Parameter	V.Th	V.Sp	Conn.D	Regression statistics
Quasi-static	k	76.49	− 11.79	− 1728	R^2^ = 0.8525, p < 0.001
		< *0.001*	< *0.001*	*0.012*	
	E_A_	1.24	− 0.19	− 37.70	R^2^ = 0.8380, p < 0.001
		< *0.001*	< *0.001*	< *0.001*	
	W_S_	105.3	− 15.24	− 1915	R^2^ = 0.8652, p < 0.001
		< 0.001	< 0.001	0.045	
Impact	F_impact_	214.7	98.99	–	R^2^ = 0.9701, p < 0.001
		< *0.001*	< *0.001*	–	
	E_S_ =	1.32	0.18	24.26	R^2^ = 0.9907, p < 0.001
		< 0.001	< 0.001	0.004	
	W_S_	26.20	48.80	7644	R^2^ = 0.9643, p < 0.001
		0.065	< 0.001	< 0.001	

The dependent variables were the stiffness (k), energy stored (E_S_), the specific energy stored (W_S_), and the impact force (F_Impact_). The independent variables were velar thickness (V.Th), velar spacing (V.Sp), and connectivity density (Conn.D). The p-values for each regression coefficient are shown in italics.

## Discussion

The mechanical performance of velar bone mimics was investigated to determine candidacy as a novel running midsole architecture. Previous studies of bighorn sheep have established that velar architecture stores energy during quasi-static^5^ and impact^6^ loading. However, there have not been any previous attempts to mimic this structure as has been done for other natural impact resistant and energy-storing materials such as nacre^12–18^, mantis shrimp dactyl club^19,20^, woodpecker skull^21^, conch shell^22^, and beetle shell^23^. Our results show that velar architecture exhibits similar bone volume fraction, larger velar thickness and spacing, and lower velar number and connectivity density compared to the analogous architectural indices in human and sheep trabecular bone. This knowledge was used to design a novel biomimetic architecture that was mechanically tested and compared to EVA running shoe midsole foams. Through an iterative design process, we developed two velar bone mimics which had greater stiffness and higher energy storage and similar specific energy stored during quasi-static compression compared to EVA foams. Additionally, the velar bone mimics had comparable impact forces to the EVA foams during dynamic impact testing; however, the mimics underperformed in energy storage and specific energy storage relative to EVA foams. These results support the potential use of incorporating velar bone bioinspired architecture into additively manufactured footwear used for quasi-static activities.

One limitation of this study is the difference in geometry between the EVA foams and the velar bone mimics. Velar bone mimics are an open-cell foam whereas the EVA foams are closed-cell. Trapped air has been shown to increase stiffness in closed-cell foams. Additionally, it is unclear that downscaling the horncore porous bone architecture to a size scale suitable for shoe soles will provide the same energy storing properties the bone structure has adapted for. However, our study is valuable because our results indicate that, despite the difference in geometry, velar bone mimics show comparable stiffness and energy storage to EVA foams in quasi-static compression. Another limitation of this study is visual isolation of the second-generation VBMs. This method was used since Abaqus does not provide utility to physically crop sections from the mesh for mimic preparation. Despite this limitation, the second-generation VBMs showed improved stiffness and energy storage over the first-generation VBMs. An additional limitation of this study is the small number of mature rams studied. Skulls of mature rams are difficult to obtain, but it is possible that this modeling approach could identify better bioinspired architectures if access to more large and mature rams becomes available. A final limitation of this study is the use of material and geometric linearity in the finite element models. Though there may be some material nonlinearity, particularly in the early and late loading zones of the force–displacement curve, much of the curve is linear. This assumption was chosen to reduce computational time for this first investigation developing velar bone inspired material architectures.

Velar bone architectural indices from the compressive region of the horncore were quantified and compared to human and sheep trabecular bone. Though many male bovids such as ibex, sheep, bison, buffalo, and antelope^3,4,65,66^ participate in intraspecific combat using their horns, the unique bone architectures present in their horns have not been studied. Velar architectural indices were compared to trabecular bone architectural indices from humans and sheep trabecular bone that experience primarily compressive loading. Our results show that the velar architecture bone volume fraction is similar to trabecular bone volume fraction in humans and sheep. However, in velar bone we found a 9-fold larger thickness, 14-fold larger spacing, 9000-fold smaller connectivity density, and 7-foldsmaller number as compared to the analogous trabecular indices in human and sheep. These differences raise the possibility that velar architecture evolved to be mechanically different from other porous bone architectures, while maintaining similar levels of bone material. However, until now, horncore biomimicks had not been investigated. Other impact-resistant biomaterials have inspired modeling and additive manufacturing of novel material architectures include ice-templating of nacre^13^, Voronoi pattern generation inspired by nacre^15^, and conch shell mimics^22^. A recent study found that, during impact, the velar bone horncore stored 3 × more strain energy than the keratin-based horn sheath^6^. Here, we showed that velar bone mimics could be additively manufactured with EPU and provide comparable energy storage in quasi-static loading to EVA foams.

Velar bone mimics were shown to have similar stiffness and energy storage during quasi-static compression compared to the EVA foams. For VBM2-2G and VBM3-2G the high energy storage and high specific energy storage can be attributed to the geometry and size of the unit cell. These specimens were made from a larger number of small unit cells as compared to the VBM1-, VBM4-, and VBM5-2G mimics. These smaller unit cells were comprised of shorter thicker velae. These thicker velae increased the stiffness of the mimic, which in turn lead to increased energy storage (i.e. larger area under the force–displacement curve). Furthermore, the stiffness of most velar bone mimics were within the range of previously measured midsole stiffnesses (30–429 N/mm^38,43–45^). These values include previous studies that tested midsoles from different manufacturers, different test geometries (whole shoe or midsole section), different materials (PU, EVA, or EPS), and with different mechanical testing procedures (displacement versus load-controlled compression). For energy storage, our results are in the range of previously published values for energy storage of midsole foams during quasi-static compression (960–1680 mJ) ^38^. However, for dynamic impact testing the energy storage and specific energy storage of the velar bone mimics is inferior to that of the EVA foams. These differences may possibly be attributed to a variety of factors such as the EPU 40 density and intrinsic energy storage, volume fraction of EPU 40, and open cell nature of the bioinspired architecture. It has been suggested that elastic compression of trapped air within a closed cell foams like EVA contributes to approximately 22% of the stiffness and 28% of the energy stored at 25% strain during compression^67^. In summary, our findings suggest the bioinspired velar mimics produced in this study may be suitable for use in additively manufactured footwear for quasi-static activities, but not for dynamic activities like running. However, with additional bighorn sheep samples, further design iterations, and improvements in additive manufacturing technologies, the dynamic performance of velar bone mimics could be improved.

Peak impact force during running is intrinsically linked to midsole stiffness^44,68^. Contradicting studies suggest negative^69^, no^70^, and positive^44,71^ correlation between midsole stiffness and impact force. Our VBM data demonstrated impact force was negatively correlated with stiffness below ~ 60 N/mm similar to the relationship for EVA foams, but for VBMs with stiffness above ~ 80 N/mm, impact force was positively correlated with stiffness (Fig. 11). . This implies that impact force can be minimized between 60 and 80 N/mm. However, even the stiffest second generation VBMs maintained impact forces within the range of the EVA foams (Fig. 10). The stepwise regression results have implications for possible future design modifications. For example, the specific energy absorbed during impact testing was positively correlated with connectivity density (Table 2). Future design approaches could consider velar bone mimics with more highly interconnected struts to improve specific energy absorption during impact. Furthermore, the length, width, and curvature of velae likely affect the mechanical behavior of the mimics, but have not yet been quantified. These parameters could also be incorporated into future design approaches for enhancing energy absorption in velar bone mimics.

In summary, we developed an iterative design process for generating novel bioinspired architectures by additive manufacturing for energy absorption applications. While some of the second-generation mimics compared similarly to EVA foam in quasi-static testing, they underperformed in dynamic testing. The bioinspired design process described here could be modified for enhanced dynamic performance in subsequent studies. In the future, similar methodology could be used to guide further development of bighorn sheep velar bone bioinspired energy storing material architectures for other applications such has helmets, packing, and vehicle panels.

## Data Availability

All data generated or analyzed during this study are included in this published article.
